# Factors Associated With Nursing Home Residents’ COVID-19 Infections: A Systematic Review of Literature

**DOI:** 10.1093/geroni/igab046.057

**Published:** 2021-12-17

**Authors:** Cheng Yin, Xiaoli Li, Rongfang Zhan, Liam ONeill

**Affiliations:** 1 University of North Texas, Corinth, Texas, United States; 2 University of North Texas, denton, Texas, United States; 3 University of North Texas, University of North Texas, Texas, United States

## Abstract

Background: Nursing homes were impacted disproportionately by the coronavirus because of their resident’s vulnerabilities and settings. Even many previous studies illustrated factors related to nursing home residents’ Covid-19 infections, there’s no such study epitomizing those factors systematically, while some factors were controversial in different studies. The article aims to summarize major types of factors and provide crucially influential implications for nursing homes to prevent and manage their resident infections. Methods: All articles published between 01 January 2020 - 15 January 2021 in English version were searched through three electronic databases (PubMed, Web of Science, and Scopus). Two authors screened and evaluated a total of 121 studies independently based on selection and extraction criteria. Results: Seventeen identified studies were included in the research, which involved five major types of factors (nursing home’s residence, nursing home, staff, resident, and others). Conclusion: nursing home’s county infection rate, size, and staff residence were the strongest significant factors in many studies. Per-capital income, symptom-based screening and testing, and asymptomatic individuals have impacted resident’s infections variously since the beginning of the pandemic. Nursing home’s star rating and a total count of fines became factors when considered its locations. Other factors, including nursing home’s type, historical health deficiencies, staffing level, and staff working different facilities, etc., were also significant factors. The value of factors suggests healthcare systems reflect appropriate measures and allocate more resources to nursing homes in high prevalence counties on the basis of universal allocation.

